# si-RNA inhibition of brain insulin or insulin-like growth factor receptors causes developmental cerebellar abnormalities: relevance to fetal alcohol spectrum disorder

**DOI:** 10.1186/1756-6606-4-13

**Published:** 2011-03-28

**Authors:** Suzanne M de la Monte, Ming Tong, Nathaniel Bowling, Peter Moskal

**Affiliations:** 1 Department of Pathology and Division of Neuropathology, Rhode Island Hospital, 593 Eddy Street, Providence, RI 02903 USA; 2 Department of Neurology, Rhode Island Hospital, 593 Eddy Street, Providence, RI 02903 USA; 3 Department of Medicine and Division of Gastroenterology, Rhode Island Hospital, 593 Eddy Street, Providence, RI 02903 USA; 4 Liver Research Center, 55 Claverick Street, Providence, RI 02903 USA; 5 Alpert Medical School at Brown University, 97 Waterman Street, Providence, RI 02912 USA; 6 Department of Biology, Brown University, 45 Prospect Street, Providence, RI 02912

## Abstract

**Background:**

In experimental models of fetal alcohol spectrum disorder (FASD), cerebellar hypoplasia and hypofoliation are associated with insulin and insulin-like growth factor (IGF) resistance with impaired signaling through pathways that mediate growth, survival, plasticity, metabolism, and neurotransmitter function. To more directly assess the roles of impaired insulin and IGF signaling during brain development, we administered intracerebroventricular (ICV) injections of si-RNA targeting the insulin receptor, (InR), IGF-1 receptor (IGF-1R), or IGF-2R into postnatal day 2 (P2) Long Evans rat pups and examined the sustained effects on cerebellar function, structure, and neurotransmitter-related gene expression (P20).

**Results:**

Rotarod tests on P20 demonstrated significant impairments in motor function, and histological studies revealed pronounced cerebellar hypotrophy, hypoplasia, and hypofoliation in si-InR, si-IGF-1R, and si-IGF-2R treated rats. Quantitative RT-PCR analysis showed that si-InR, and to a lesser extent si-IGF-2R, broadly inhibited expression of insulin and IGF-2 polypeptides, and insulin, IGF-1, and IGF-2 receptors in the brain. ELISA studies showed that si-InR increased cerebellar levels of tau, phospho-tau and β-actin, and inhibited GAPDH. In addition, si-InR, si-IGF-1R, and si-IGF-2R inhibited expression of choline acetyltransferase, which mediates motor function. Although the ICV si-RNA treatments generally spared the neurotrophin and neurotrophin receptor expression, si-InR and si-IGF-1R inhibited NT3, while si-IGF-1R suppressed BDNF.

**Conclusions:**

early postnatal inhibition of brain InR expression, and to lesser extents, IGF-R, causes structural and functional abnormalities that resemble effects of FASD. The findings suggest that major abnormalities in brains with FASD are mediated by impairments in insulin/IGF signaling. Potential therapeutic strategies to reduce the long-term impact of prenatal alcohol exposure may include treatment with agents that restore brain insulin and IGF responsiveness.

## Background

In the central nervous system (CNS), insulin and insulin-like growth factors (IGFs) transmit pro-growth and pro-survival signals by activating complex intracellular pathways, beginning with ligand binding to cell surface receptors. Activated receptor tyrosine kinases phosphorylate insulin receptor substrate (IRS) proteins [1], which then interact with *src *homology domain-containing adaptor molecules to promote mitogenesis, cell survival, gene expression, metabolism, and motility [1-3]. Insulin, IGF-1 and IGF-2, and their corresponding receptors are abundantly expressed in neurons [1,4,5] and glia [6-8] throughout the brain, but the highest levels are distributed in the hypothalamus, temporal lobe, and cerebellum [1], i.e. major targets of ethanol-mediated neurotoxicity. Given that insulin and IGF mediate neuronal survival, plasticity, energy metabolism, and neurotransmitter function [1,9-12], we hypothesized that sustained impairments in these signaling pathways would have dire consequences with respect to CNS function.

During development, the brain is a major target of alcohol-mediated toxicity and teratogenesis. The constellation of neurodevelopmental and behavioral abnormalities caused by chronic gestational exposure to alcohol is termed, "fetal alcohol spectrum disorder" (FASD) [13-19]. In the cerebellum, effects of FASD include hypoplasia, hypo-foliation, reduced neuronal survival, disordered cell migration, and impaired motor function [15-17,20-22]. There is now sufficient evidence that many of these adverse effects of ethanol are mediated by impairments in insulin and IGF signaling [22-27]. Ethanol impairs insulin and IGF signaling in immature neuronal cells [28] by reducing ligand-receptor binding, and inhibiting receptor tyrosine kinase activation of downstream pathways through IRS [22-27]. Consequences include, down-regulation of insulin/IGF responsive genes [22,24,26,29,30], increased oxidative stress, DNA damage, lipid peroxidation, and mitochondrial dysfunction [24,25,31,32], and reduced neuronal survival, plasticity [24,32,33], energy metabolism, and choline acetyltransferase (ChAT) activity [22,25,27,34]. ChAT is a major neurotransmitter that is needed for cognitive and motor functions [35-38].

The present study examines the degree to which inhibition of insulin/IGF signaling through their corresponding receptors is sufficient to mimic ethanol-impaired cerebellar development and function. We utilized an in vivo model in which insulin or IGF receptor genes were inhibited in rat pup brains by intracerebroventricular (ICV) delivery of si-RNA duplexes targeting the insulin receptor (si-InR), IGF-1 receptor (si-IGF-1R), or IGF-2 receptor (si-IGF-2R), or nonspecific sequences (si-Scr) [39,40], and examined the sustained effects of these si-RNA treatments on motor function, cerebellar structure, and neurotransmitter and neurotrophin gene expression. The cerebellum was the focus of our investigations because: 1) it develops mainly in the early postnatal period; 2) it is one of the primary targets of ethanol-mediated neurotoxicity (other sites include the hippocampus, corpus callosum, cerebral cortex, and caudate nucleus); and 3) it expresses abundant levels of insulin and IGF receptors during development [1,20,22,27,41-43].

## Methods

### Materials

Qiazol reagent, EZ1 RNA universal tissue kit, QuantiTect SYBR Green polymerase chain reaction (PCR) master mix, and the BIO Robot Z1 were from Qiagen Inc (Valencia, CA). Monoclonal antibodies to β-Actin, tau, phospho-tau (AT8-S199, S202, T205), brain-derived neurotrophic factor (BDNF), nerve growth factor (NGF), neurotrophin 3 (NT3), neurotrophin 4 (NT4), glyceraldehyde-3-phosphate dehydrogenase (GAPDH), choline acetyltransferase (ChAT), acetylcholinesterase (AChE), and 4-hydroxy-2-nonenal (HNE) were purchased from Abcam Inc. (Cambridge, MA), Santa Cruz Biotechnology Inc. (Santa Cruz, CA), or Chemicon International (Tecumsula, CA). The 85G6 aspartyl-(asparaginyl)-β-hydroxylase (AAH) monoclonal antibody was generated to human recombinant protein and purified over Protein G columns (Healthcare, Piscataway, NJ) [44]. Small interfering RNA duplex molecules and Dharmafect reagent were purchased from Dharmacon, Inc. (Chicago, IL). Histofix was purchased from Histochoice (Amresco, Solon, OH). The AMV first strand cDNA synthesis kit was obtained from Roche Diagnostics Corporation (Indianapolis, IN). Diaminobenzidine (DAB) chromogen was from Vector Laboratories (Burlingame, CA). PCR primers were purchased from Sigma Chemical Co. (St. Louis, MO). Enzyme-linked immunosorbant assay (ELISA) 96-well plates and the ELISA plate washer were from Nunc (Rochester, NY). Horseradish peroxidase (HRP)-conjugated secondary antibody and Amplex Red soluble fluorophore were from Invitrogen (Carlsbad, CA). HRP-labeled polymer conjugated secondary antibody used for immunohistochemistry was purchased from Dako Corp (Carpinteria, CA). The SpectraMax M5 microplate reader was purchased from Molecular Devices Corp. (Sunnyvale, CA). NanoOrange and the bicinchoninic acid (BCA) assay reagents were from Pierce chemical Corp. (Rockford, IL). All other fine chemicals were purchased from CalBiochem (Carlsbad, CA), Pierce (Rockford, IL), or Sigma (St. Louis, MO).

### Gene Delivery Model

Postnatal day 2 (P2) Long Evans rat pups were given a single intracerebroventricular (ICV) injection of small interfering RNA duplexes (si-RNA) targeting the insulin receptor (si-InR) [INSR NM_017071], IGF-1 receptor (si-IGF-1R) [IGF1R NM_052807], IGF-2R (si-IGF-2R) [IGF2R NM_012756], or nonspecific sequences (Scrambled; si-Scr) [NM D-001210-01-20]. For each rat, 0.4 nmol si-RNA plus 100 ng recombinant green fluorescent protein (GFP) expressing plasmid were complexed with 10 μl of Dharmafect reagent, and injected into the right frontal region over the lateral ventricle using a Hamilton syringe with a 26-gauge needle as previously described [39,40]. GFP expression, which was under the control of a CMV promoter [45], was used to monitor success of transfection. This was accomplished by sacrificing rats (N = 4 per group) at regular intervals after the ICV gene delivery, and measuring GFP mRNA levels in whole brain homogenates by qRT-PCR analysis. Those studies demonstrated peak levels of GFP expression within 2-3 days after ICV gene delivery, followed by a gradual decline in GFP expression. Importantly, GFP expression persisted over the time course of the experiment, consistent with findings in previous studies [39]. All rats survived the procedure, and none exhibited adverse responses such as, failure to thrive, poor grooming, reduced physical activity, or weight loss.

Gene expression and histological studies pertaining to the effects of si-InR, si-IGF-1R, or si-IGF-2R were performed with cerebella harvested on P24. Upon sacrifice, freshly harvested cerebella were divided in the mid-sagittal plane. One hemisphere was immersion fixed in Histofix, and the other was snap-frozen in a dry ice/methanol bath and stored at -80°C for mRNA and protein studies. The fixed tissue was embedded in paraffin and used to generate hematoxylin and eosin (H&E) stained histological sections (5 μm-thick). In addition, adjacent sections were immunostained with monoclonal antibodies to 4-hydroxy-2-nonenal (HNE) or aspartyl-(asparaginyl)-β-hydroxylase (AAH), as previously described [44], except that immunoreactivity was detected with HRP-labeled polymer-conjugated secondary antibody and DAB. The sections were lightly counterstained with Hematoxylin and examined by light microscopy. Our protocol was approved by the Institutional Animal Care and Use Committee at Lifespan-Rhode Island Hospital, and it conforms to the guidelines set by the National Institutes of Health.

### Rotarod Testing

We used rotarod testing to assess long-term effects of ICV si-InR, si-IGF-1R and si-IGF-2R treatments on motor function [46,47]. On P19, rats were trained to remain balanced on the rotating Rotamex-5 apparatus (Columbus Instruments) at 1-5 rpm. On P20, rats (N = 8-10 per group) were administered 10 trials at incremental speeds up to 10 rpm, with 10 minutes rest between each trial. The latency to fall was automatically detected and recorded with photocells placed over the rod. However, trials were stopped after 30 seconds to avoid exercise fatigue. Data from trials 1-3 (2-5 rpm), 4-7 (5-7 rpm), and 8-10 (8-10 rpm) were culled and analyzed using the Mann-Whitney test.

### Quantitative Reverse Transcriptase Polymerase Chain Reaction (qRT-PCR) Analysis

We used qRT-PCR to measure mRNA expression as previously described [48-50]. In brief, cerebella were homogenized in Qiazol reagent, and total RNA was isolated using the EZ1 RNA universal tissue kit and the BIO Robot EZ1. RNA was reverse transcribed using random oligodeoxynucleotide primers and the AMV First Strand cDNA synthesis kit. The resulting cDNA templates were used in qPCR amplification reactions with gene specific primer pairs (Additional File 1) [51]. Primers were designed using MacVector 10 software (MacVector, Inc., Cary, NC) and their target specificities was verified using NCBI-BLAST (Basic Local Alignment Search Tool). The amplified signals were detected and analyzed using the Mastercycler ep realplex instrument and software (Eppendorf AG, Hamburg, Germany). Relative mRNA abundance was calculated from the ng ratios of specific mRNA to 18S rRNA measured in the same samples. Assays were performed in triplicate. Inter-group statistical comparisons were made using the calculated mRNA/18S ratios.

### Enzyme Linked Immunosorbent Assay (ELISA)

Cerebellar homogenates were prepared in radioimmunoprecipitation assay (RIPA) buffer containing protease and phosphatase inhibitors [50]. Protein concentrations were determined using the bicinchoninic (BCA) assay. We performed direct binding ELISAs to measure immunoreactivity. Samples containing 50 ng protein diluted in Tris buffered saline, pH 7.4 (TBS) were adsorbed to the bottom flat surfaces of 96-well polystyrene ELISA plates overnight at 4°C [44]. Non-specific binding sites were blocked by a 3-hour room temperature incubation with 300 μl/well of TBS + 0.05% Tween 20 + 3% BSA. Samples were then incubated with 0.1-0.5 μg/ml primary antibody for 1 h at 37°C. Immunoreactivity was detected with horseradish peroxidase (HRP)-conjugated secondary antibody and Amplex Red soluble fluorophore [44]. Fluorescence was measured (Ex 530/Em 590) in a SpectraMax M5 microplate reader. Parallel negative control assays had primary, secondary, or both antibodies omitted. Between steps, reactions were rinsed 3 times with TBS + 0.05% Tween 20 using a Nunc ELISA plate washer. Levels of immunoreactivity were normalized to protein content in the wells, which was measured using NanoOrange reagent.

### Statistical Analysis

Data corresponding to levels of gene expression or immunoreactivity are depicted in boxplot graphs representing the means (horizontal bars), 95% confidence intervals (box limits), and range (whiskers) for each group. Inter-group comparisons were made using the Kruskal-Wallis one-way Analysis of variance (ANOVA) with the Dunn's multiple comparisons post-hoc test of statistical significance. Statistical analyses were performed using the GraphPad Prism 5 software (San Diego, CA) and significant P-values (<0.05) are indicated within the graph panels.

## Results

### ICV injection of si-RNA targeting the insulin or IGF receptors impairs motor performance

**Figure 1:**
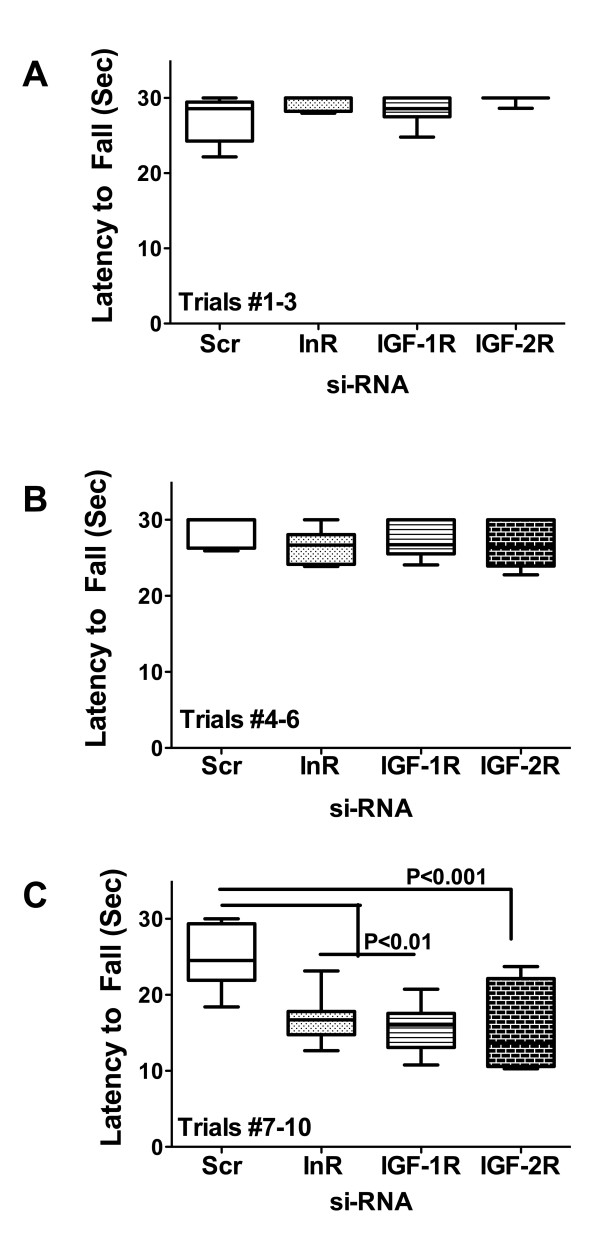
**Effects of si-InR, si-IGF-1R, and si-IGF-2R ICV injections on motor performance**. P2 Long Evans rat pups were administered intracerebroventricular (ICV) injections of si-RNA targeting the insulin receptor (InR), insulin-like growth factor type 1 receptor (IGF-1R), IGF-2R, or nonspecific sequences (Scr). On P20, rats were subjected to 10 incremental speed trials (from 2 to 10 rpm) of rotarod testing of motor function. Each trial was limited to 30 seconds duration. Data from (A) Trials 1-3 (2-4.5 rpm), (B) Trials 4-6 (5-7.5 rpm), and (C) Trials 7-10 (8-10 rpm) were culled and analyzed using the Mann-Whitney test. Panels display box plots with means (bar), 95% confidence limits (box) and range (whiskers). Significant P-values are indicated within the panels.

Rotarod tests are used to assess sensorimotor coordination and they provide a highly sensitive index of damage to the cerebellum [46,52]. Rotarod test results were analyzed by grouping performance for Trials 1-3, 4-6, and 7-10, in which the rotation speeds were incremented from 2 to 4.5, 5 to 7.5, and 8 to 10 rpm, respectively. The mean ± S.E.M. latency to fall periods were calculated and results are depicted graphically. In the two lower speed trial sets, all 4 groups performed similarly (Figures 1A-1B), whereas in the final and most challenging trial series, rotarod performance was significantly impaired in the si-InR, si-IGF-1R, and si-IGF-2R relative to si-Scr treated controls (Figure 1C).

### Cerebellar hypofoliation and hypotrophy following ICV injection of si-InR, si-IGF-1R, or si-IGF-2R

**Figure 2:**
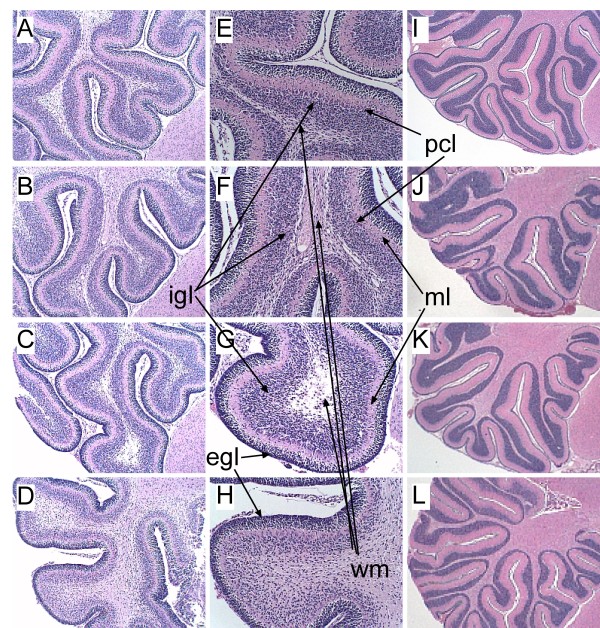
**Intracerebroventricular si-InR, si-IGF-1R, or si-IGF-2R impairs cerebellar development**. Cerebella of rats treated by intracerebroventricular (ICV) injection of si-RNA targeting (A,E,I) nonspecific sequences (Scr), (B,F,J) InR, (C,G,K) IGF-1R, or (D,H,L) IGF-2R, were harvested on (A-H) P9 or (I-L) P20, sectioned in the mid-sagittal plane, fixed in Histochoice, and embedded in paraffin. Histological sections were stained with Hematoxylin and Eosin. Photographs were taken at magnifications of (A-D) 100×, (E-H) 200×, or (I-L) 40×. (B-D) Note relative simplification of the folia and variability in the thickness of the cortex, (D, H) reduced compactness and cellularity within the internal granule cell layer, and (B-D, F-H) impaired development of white matter (wm) with (B,F,D,H) increased gliosis or (C,G) markedly reduced parenchymal volume and appearance of cavitation one week after the ICV injections of si-Inr, si-IGF-1R, or si-IGF-2R relative to si-Scr controls (A,E). (I-L) In P20 rats, the structural differences in cerebellar architecture caused by the si-RNA treatments were more subtle and mainly manifested by slightly more irregular and shallow folia in (J) si-InR and (L) si-IGF-2R treated relative to (I) si-Scr controls. Abbreviations: igl = internal granule cell layer; egl = external granule cell layer; pcl = Purkinje cell layer; wm = white matter; ml = molecular layer.

Cerebella were harvested to examine histopathological effects of the ICV siRNA treatments, on P9 and P16 (Figure 2). Cerebella from si-Scr transfected controls had well-developed complex foliation with regular patterns of major deep and minor shallow grooving, slender white matter cores, compact and densely populated granule and Purkinje cell layers, and a uniform molecular layer (Figures 2A, 2E, 2I). In contrast, P9 brains, including cerebella from si-InR, si-IGF-1R, or si-IGF-2R-transfected rats were smaller, and they had relatively simplified, shallow, blunted, and irregular folia, irregularly thickened white matter cores, and conspicuously hypocellular cortical granule cell layers relative to control (Figures 2B-2D, 2F-2H). The si-IGF-2R transfected rats had the most pronounced simplification and hypotrophy of cerebellar folia compared with all other groups (Figure 2). It is noteworthy that the Purkinje layer of cortex was consistently preserved, whereas the granule cell and molecular layers were variably affected by the si-RNA treatments. The relative sparing of Purkinje cells was most likely due to the fact that they develop in utero, while the granule and molecular layers develop mainly within the early postnatal period [42,43,53] during which the ICV si-RNAs were delivered. At the later time point (P16), the effects of the siRNA treatments on cerebellar structure were subtler, but still manifested by relatively simplified and shallow foliation relative to control (Figures 2I-2L).

Adjacent histological sections were immunostained with monoclonal antibodies to HNE to assess levels of oxidative stress and lipid peroxidation, or AAH, which is a downstream target of insulin/IGF signaling and regulator of neuronal migration [44,45] (Additional File 2). Previous studies demonstrated that chronic in utero ethanol exposure increases HNE and reduces AAH immunoreactivity [22,44,54]. In the present study, si-Scr treated control cerebella had low levels of HNE immunoreactivity, while cerebella exposed to specific si-InR, si-IGF-1R, or si-IGF-2R had increased levels HNE immunoreactivity localized in the granule cell and Purkinje cell layers, as well as in white matter glia. ICV injection of si-IGF-2R increased HNE immunoreactivity to a greater extent than the other si-RNA treatments. The si-Scr treated control cerebella had abundant AAH immunoreactivity distributed in Purkinje and granule cells, as well as in neuropil fibrils. In contrast, ICV transfection with si-RNA targeting InR, IGF-1R, or IGF-2R was associated with lower levels of cerebellar AAH immunoreactivity relative to control. However, si-InR treatment resulted in increased AAH immunoreactivity in reactive, hypertrophic astrocytes distributed among granule cells.

### ICV injection of si-InR, si-IGF-1R, or si-IGF-2R inhibits cerebellar insulin, IGF-1, IGF-2 polypeptide and receptor gene expression

**Figure 3:**
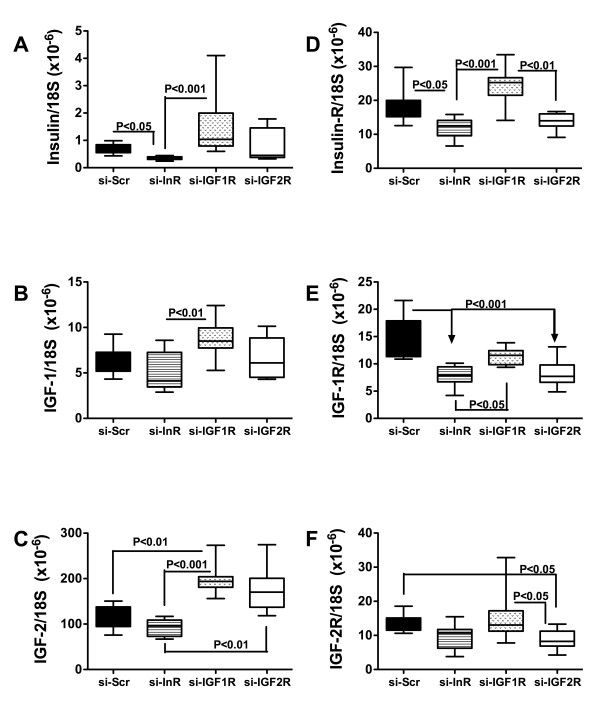
**Inhibition of insulin and IGF signaling mechanisms in cerebella following ICV si-RNA delivery**. Long Evans rat pups were administered ICV injections of si-Scr, si-InR, si-IGF-1R, or si-IGF-2R on P2. Cerebella harvested on P24 were used to measure (A) insulin, (B) IGF-1, (C) IGF-2, (D) insulin R, (E) IGF-1R, and (F) IGF-2R expression by qRT-PCR analysis with results normalized to 18S rRNA. Box plots depict means, 95% confidence intervals, and range for each group. Inter-group statistical comparisons were made using the Kruskal-Wallis one-way ANOVA and the Dunn's multiple comparisons post hoc test. Significant P-values are indicated within the panels.

ICV injection of si-InR significantly reduced the mean mRNA levels of insulin, insulin receptor, and IGF-1R relative to control. In addition, si-InR treated cerebella had significantly lower mean levels of IGF-1, IGF-2, insulin receptor, and IGF-1R relative to si-IGF-1R treated rats, and lower levels of IGF-2 compared with si-IGF-2R treated rats (Figure 3). Rats treated by ICV injection of si-IGF-1R had significantly higher cerebellar levels of IGF-2 expression relative to control, higher levels of insulin, IGF-1, IGF-2, insulin receptor, and IGF-1R relative to si-InR treated rats, and higher levels of IGF-2R relative to si-IGF-2R treated rats. ICV injection of si-IGF-2R significantly reduced IGF-1R and IGF-2R expression relative to control.

### si-InR, si-IGF-1R, and si-IGF-2R alter neurotrophin and neurotrophin receptor expression in brain

**Figure 4:**
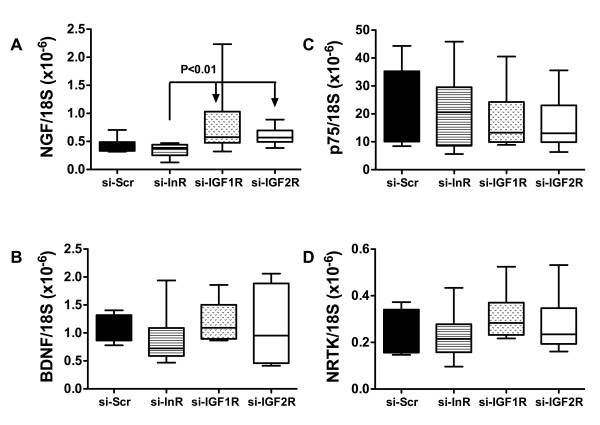
**Effects of ICV administration of si-RNA targeting insulin or IGF receptors on neurotrophin and neurotrophin receptor expression**. Long Evans rat pups were administered ICV injections of si-Scr, si-InR, si-IGF-1R, or si-IGF-2R on P2. Cerebella harvested on P24 were used to measure (A) NGF, (B) BDNF, (C) p75, and (D) NTRK expression by qRT-PCR analysis with results normalized to 18S rRNA. Box plots depict means, 95% confidence intervals, and range for each group. Inter-group statistical comparisons were made using the Kruskal-Wallis one-way ANOVA and the Dunn's multiple comparisons post hoc test. Significant P-values are indicated within the panels.

**Figure 5:**
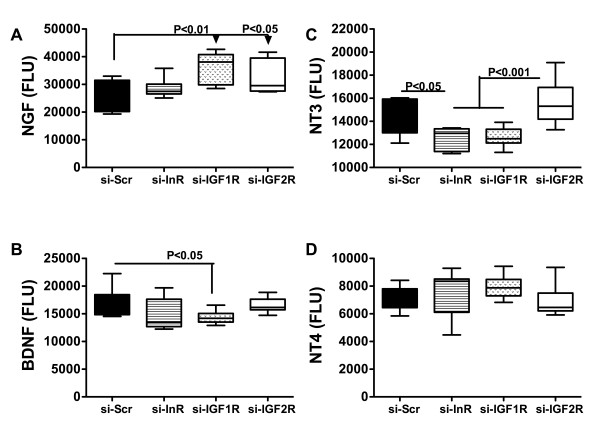
**Effects of ICV injection of si-RNA targeting insulin or IGF receptors on neurotrophin immunoreactivity**. Long Evans rat pups were administered ICV injections of si-Scr, si-InR, si-IGF-1R, or si-IGF-2R on P2. Cerebella harvested on P24 were used to measure (A) NGF, (B) BDNF, (C) NT3, and (D) NT4 immunoreactivity by ELISA, with results normalized to protein content within the wells. Immunoreactivity was detected with horseradish peroxidase conjugated secondary antibody and the Amplex Red fluorophore. Fluorescence was measured in a Spectramax M5 microplate reader (Ex 530 nm/Em 590 nm). Box plots depict means, 95% confidence intervals, and range for each group. Inter-group statistical comparisons were made using the Kruskal-Wallis one-way ANOVA and the Dunn's multiple comparisons test. Significant P-values are indicated within the panels.

We used qRT-PCR analysis (Figure 4) and ELISAs (Figure 5) to measure expression of nerve growth factor, brain derived neurotrophic factor (BDNF), p75 and neuronal tyrosine (NRTK) kinase receptors in cerebellar tissue (Figure 4). The qRT-PCR studies demonstrated significantly lower mean levels of NGF in si-InR treated relative to si-IGF-1R and si-IGF-2R treated rats. Otherwise, the mRNA levels of BDNF, p75 and NRTK were similar among the groups. ELISA studies (Figure 5) revealed significant reductions in NT3 and BDNF neurotrophin immunoreactivity in si-InR and/or si-IGF-1R treated, but not si-IGF-2R treated cerebella relative to control. The mean levels of NGF were similar in the control and si-InR treated groups, but significantly elevated relative to control in si-IGF-1R and si-IGF-2R treated rats. The levels of NT3 immunoreactivity were similar in control and si-IGF-2R treated rats, but significantly reduced relative to control or si-IGF-2R in both si-InR and si-IGF-1R treated rats. The mean levels of BDNF immunoreactivity were similar in control, si-InR, and si-IGF-1R treated rats, but significantly reduced in the si-IGF-1R treated relative to control rats. In contrast, no significant inter-group differences were observed with respect to NT4 expression.

### Inhibition of insulin or IGF receptors alters tau, pTau, ChAT, and GAPDH expression

**Figure 6:**
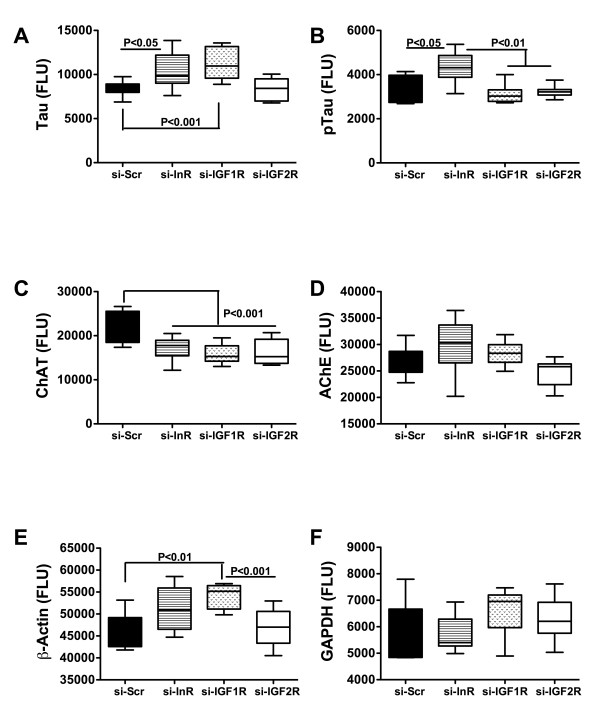
**Effects of ICV injection of si-RNA targeting insulin or IGF receptors on indices of neuronal structure and function**. Long Evans rat pups were administered ICV injections of si-Scr, si-InR, si-IGF-1R, or si-IGF-2R on P2. Cerebella harvested on P24 were used to measure (A) Tau, (B) phospho-Tau; pTau), (C) choline acetyltransferase; ChAT (D) acetylcholinesterase; AChE, (E) β-Actin, and (F) GAPDH immunoreactivity by ELISA with results normalized to protein content within the wells. Immunoreactivity was detected with horseradish peroxidase conjugated secondary antibody and the Amplex Red fluorophore. Fluorescence was measured in a Spectramax M5 microplate reader (Ex 530 nm/Em 590 nm). Box plots depict means, 95% confidence intervals, and range for each group. Inter-group statistical comparisons were made using the Kruskal-Wallis one-way ANOVA and the Dunn's multiple comparisons test. Significant P-values are indicated within the panels.

Brain insulin/IGF resistance is associated with accumulation of tau and phospho-tau, inhibition of cholinergic function, and impairments in energy metabolism [22,24,41,54,55]. ELISAs were used to measure tau, phospho-tau (pTau), ChAT, AChE, β-Actin, and GAPDH immunoreactivity (Figure 6). Both si-InR and si-IGF-1R significantly increased tau immunoreactivity, while si-InR significantly increased pTau, and si-IGF-1R significantly increased β-Actin. ICV delivery of si-InR, si-IGF-1R, and si-IGF-2R significantly reduced ChAT immunoreactivity relative to control, but did not significantly alter the mean levels of AChE immunoreactivity. Finally, si-InR, but not si-IGF-1R or si-IGF-2R ICV treatment reduced GAPDH expression in brain.

## Discussion

Humans and experimental animals afflicted with FASD have sustained impairments in cognitive and motor functions. With regard to motor functions, the cerebellum is a major target of alcohol's neurotoxic and teratogenic effects during development. Phenotypic manifestations of FASD in the cerebellum include hypoplasia, hypofoliation, and disordered patterns of neuronal migration [15-21,56,57]. These effects of prenatal ethanol exposure are associated with impairments in neuronal growth, survival, motility, adhesiveness, energy metabolism, mitochondrial function, plasticity, and neurotransmitter function, and increased oxidative stress and DNA damage [22,25-27,42,54,58-67].

Ethanol mediates its adverse effects on CNS neurons through inhibition of insulin and insulin-like growth factor (IGF) signaling [34,68,69]. Ethanol impairs binding to the insulin/IGF receptors, preventing activation of receptor tyrosine kinases that transmit signals downstream through IRS molecules [22-28]. In addition, ethanol inhibits other kinases, and it activates phosphatases at various levels within the insulin/IGF signaling cascades [27,70]. The net result is to reduce the levels and duration of positive signaling. While these effects of ethanol support our hypothesis that several fundamental CNS abnormalities in FASD are attributable to impairments in insulin and IGF signaling, they do not exclude a role for neurotoxic injury caused by ethanol or its metabolites. In this regard, it is noteworthy that oxidative stress activates pro-inflammatory cytokines, which promote cellular injury, DNA damage, mitochondrial dysfunction, cell death, and insulin resistance [24,25,31,32], while at the same time, insulin resistance promotes oxidative stress. The present study attempts clarify the contribution of reduced insulin and IGF signaling as a mediator of impaired cerebellar development and function as occur in FASD.

We used ICV injections to transfect immature brains with si-RNA duplex molecules targeting InR, IGF1R, IGF-2R, or nonspecific sequences (si-Scr). GFP expressing plasmid DNA was co-transfected in order to confirm success of the nucleic acid delivery. GFP expression, detected by qRT-PCR analysis, peaked within 2-3 days of gene delivery, and persisted throughout the period of study. Previously, we used the same approach to generate a model of neurodegeneration, and demonstrated sustained transgene expression throughout the brain and in all cell types [39]. However, multiple attempts to generate similar models in adult brains proved unsuccessful. One limitation of this model is the inability to truly examine dose-effects since the method of gene suppression was transient and based on delivery of si-RNA molecules. On the other hand, an advantage of this approach is that the RNA duplexes do not persist in the brain, and therefore, any long-term effects could be attributed to transient targeted disruption of gene expression during a critical period of development. Although we did not include data from un-operated controls in this manuscript, the rotarod performance and brain structure in si-Scr transfected rats were comparable to previous findings in un-operated controls.

Rotarod tests performed nearly 2 weeks after the ICV injections revealed significant impairments in motor function in the si-InR, si-IGF-1R, and si-IGF-2R relative to si-Scr controls. Correspondingly, all 3 experimental groups had conspicuous abnormalities in cerebellar structure with hypotrophy, granule cell layer thinning with hypoplasia, and hypofoliation. In addition, the white matter cores were excessively thickened in si-InR and si-IGF-1R treated rats, and markedly attenuated in si-IGF-2R treated rats. Overall structural abnormalities in the cerebellum were most pronounced in si-IGF-2R-treated rats, suggesting that signaling through the IGF-2R is critical for early postnatal cerebellar development. The finding that Purkinje cells were relatively spared corresponds with their earlier in utero development [43,53,71]. Granule cells and white matter fibers develop in the postnatal period when the si-RNA treatments were administered.

Quantitative RT-PCR analysis showed that si-InR broadly inhibited expression of insulin and IGF-2 polypeptides, and insulin, IGF-1, and IGF-2 receptors in brain. ELISA studies confirmed that the si-InR treatments inhibited expression of InR, IGF-1R, and IGF-2R. The si-IGF-2R treatments had similar but more restrictive inhibitory effects on insulin/IGF polypeptide and receptor expression in brain. These results suggest that InR is co-expressed with IGF-1R or IGF-2R and/or that insulin and IGF-2 polypeptides are expressed in InR- and/or IGF-2R-positive cells. This overlap of signaling mechanisms suggests that these related receptors serve distinct functions during development, and the co-expression of trophic factors suggests the existence of either paracrine or autocrine stimulation networks. On the other hand, impaired signaling through InR, as occurs with ethanol exposure [22,72], reduces neuronal survival, energy metabolism, neurotransmitter function, particularly cholinergic, and mitochondrial function. The attendant increased apoptosis of InR-positive cells would likely reduce the population of IGF-1R- and IGF-2R-bearing cells, and thereby decrease responsiveness to insulin, IGF-1 and IGF-2, since InR and IGF-1R or IGF-2R are likely co-expressed in developing cerebellar cells.

Reduced responsiveness to trophic factors would mimic effects of insulin/IGF resistance. In addition, since insulin and IGFs are expressed in InR, IGF-1R, and/or IGF-2R-bearing cells, trophic factor resistance and attendant cell loss would also lead to trophic factor deficiency. These points are relevant to FASD because ethanol exposure during development causes both insulin/IGF resistance and deficiency in cerebella and cerebellar granule neurons [22,27]. Moreover, the findings herein suggest that several characteristic structural and functional cerebellar abnormalities associated with FASD could be mediated by inhibition of signaling through the insulin and IGF receptors. Corresponding with observations in other models of brain insulin and IGF resistance, it may be possible to prevent or reduce the severity of ethanol-induced neurodevelopmental abnormalities with insulin sensitizer therapeutic agents such as peroxisome-proliferator activated receptor agonists [73]. Recent preliminary studies suggest that this approach would be effective for restoring cerebellar function impaired by early postnatal ethanol exposure [41].

Consequences of impaired insulin and IGF signaling include inhibition of target gene expression and post-translational modification of certain proteins. Insulin and IGF regulate expression of GAPDH [74], ChAT [75], and AAH [76]. Correspondingly, the studies herein demonstrated that siRNA targeting of InR, IGF-1R, or IGF-2R inhibited expression of all 3 proteins. In contrast, phospho-Tau, tau and β-actin levels were increased. si-InR, si-IGF-1R, and si-IGF-2R inhibition of GAPDH, which has an important role in energy metabolism, could account for the associated increased cerebellar levels of HNE. HNE, a marker of oxidative stress and lipid peroxidation, is also increased in brains afflicted with FASD and in neuronal cells exposed to ethanol in vitro [54]. ChAT, a major neurotransmitter that mediates motor function, is stimulated by insulin or IGFs, and correspondingly, ChAT expression was significantly reduced in brains transfected with si-InR, si-IGF-1R, or si-IGF-2R. This effect could account for the significant impairments in Rotarod performance observed in all 3 experimental groups. In contrast to ChAT, neurotrophin and neurotrophin receptor expression were relatively spared by the si-RNA treatments, with the exceptions of NT3, which was inhibited by si-InR and si-IGF-1R, and BDNF, which was suppressed by si-IGF-1R. Both BDNF and NT3 are inhibited by ethanol [61], and their expression and function are intimately tied to insulin and IGF signal transduction cascades [77,78].

ICV delivery of si-InR, si-IGF-1R, or si-IGF-2R reduced AAH immunoreactivity in cerebellar granule cells as demonstrated by immunohistochemical staining. AAH is another downstream target of insulin and IGF stimulation [45,76] and a mediator of cell migration [48,79]. AAH promotes cell motility by interacting with, and hydroxylating Notch and its ligand, Jagged [80]. Notch signaling activates the transcription factors, HES and HEY [81,82], which modulate expression of target genes [83]. In FASD, impaired neuronal migration and cerebellar foliation correlate with insulin/IGF resistance and reduced AAH expression [44]. Since the si-RNA treatments mimicked these effects of FASD, it is likely that ethanol's inhibition of insulin/IGF signaling is sufficient to cause these teratogenic cerebellar abnormalities.

The increased cerebellar levels of phospho-tau in si-InR treated rats is explainable on the basis of relative insulin resistance since impaired insulin signaling leads to increased activation of kinases, including GSK-3β, that promote tau phosphorylation [1]. High levels of phospho-tau can be neurotoxic and frequently are associated with neuronal injury, neurodegeneration, or oxidative stress [50,69,84-87]. On the other hand, increased levels of tau and β-actin immunoreactivity in si-InR and si-IGF-1R treated rats, respectively suggest that impaired insulin and IGF-1 signaling may result in accumulation of cytoskeletal proteins. Although the consequences of these effects are unknown, they could promote oxidative stress and thereby contribute to the observed structural and functional abnormalities in the brain.

The relevance of these findings to FASD is that, both the ICV si-InR/IGF-R and developmental (prenatal or early postnatal) exposures to ethanol cause cerebellar hypoplasia with loss of cortical granule cells [22,27], disordered cell migration in the cerebellum [22,27], and impaired performance on rotarod testing [41]. In addition, reduced expression of InR/IGF-R is associated with reduced expression of insulin and IGF polypeptide genes, GAPDH, ChAT, and AAH [22,27,44], and increased oxidative stress [22,54] in cerebella of both the si-InR/IGF-R and prenatal ethanol exposure models. Although we have not yet published the effects of ethanol on tau expression and phosphorylation in the brain, a recent study by Saito, et al showed that early postnatal exposure to ethanol leads to increased phosphorylation, cleavage, and degradation of tau due to increased GSK-3β and caspase-3 activation in the developing brain [88]. Of note is that similar mechanisms of AAH inhibition and degradation were also demonstrated in our model of FASD [45]. Therefore, the increased tau phosphorylation observed in cerebella of si-InR treated rats is also a feature of FASD. In contrast to our finding of increased β-actin immunoreactivity in cerebella of si-InR and si-IGF-1R treated rats, previous studies have demonstrated no significant effects of ethanol on β-actin expression [45], and instead showed that ethanol impairs cytoskeletal organization and adhesion mechanisms [89]. Altogether, the adverse effects of early postnatal ICV si-InR/IGF-R treatment on cerebellar development and function are similar to but less severe compared with FASD. The differences could be accounted for in part by the fact that both insulin and IGF receptors are impaired by ethanol, and that ethanol as well as acetaldehyde exert pronounced toxic and metabolic effects on immature neuronal cells [90].

## Conclusions

The principal finding in this work were that molecular inhibition of the insulin or IGF receptors in the developing brain is sufficient to produce many of the effects associated with FASD in the cerebellum, including structural abnormalities, impaired motor function, altered expression of neurotrophins, neurotropin receptors, and genes and proteins that are regulated by insulin/IGF signaling, and increased oxidative stress. The more pronounced adverse effects of ethanol relative to ICV si-InR, si-IGF-1R, or si-IGF-2R could be attributed to broad-spectrum inhibition of these receptors and corresponding trophic factors, together with more pronounced oxidative injury mediated by the toxic effects of both ethanol and its chief metabolite, acetaldehyde.

## List of abbreviations

AAH: aspartyl-(asparaginyl)-β-hydroxylase; AChE: acetylcholinesterase; ANOVA: analysis of variance; BCA: bicinchoninic assay; BDNF: brain-derived neurotrophic factor; ChAT: choline acetyltransferase; CNS: central nervous system; DAB: diaminobenzidine; ELISA: enzyme-linked immunosorbent assay; FASD: fetal alcohol spectrum disorder; GAPDH: glyceraldehyde-3-phosphate dehydrogenase; GFP: green fluorescent protein; H&E: hematoxylin and eosin; HNE: 4-hydroxy-2-nonenal; HRP: horseradish peroxidase; ICV: intracerebroventricular; IGF: insulin-like growth factor; IRS: insulin receptor substrate; NGF: nerve growth factor; NRTK: neuronal tyrosine kinase receptor; NT: neurotrophin; P: postnatal day; qRT-PCR: quantitative reverse transcriptase polymerase chain reaction; R: receptor; RIPA: radioimmunoprecipitation assay; Scr: scrambled; si: short-interfering; TBS: Tris buffered saline.

## Competing interests

The authors declare that they have no competing interests.

## Authors' contributions

SMDLM conceived of, designed, and directed the research project, analyzed the data, and wrote the manuscript. MT performed most of the in vivo experiments and molecular studies. NB assisted with gene expression analysis and gene delivery experiments, and prepared draft portions of the manuscript. PM assisted with gene expression analysis and gene delivery experiments, and prepared draft portions of the manuscript. All authors have read and approved the final manuscript.

## Supplementary Material

Additional file 1 **Primer pairs used for qRT-PCR assays**. The table includes primer pair sequences, primer binding positions on the mRNA, and the amplicon (PCR product) sizes. Click here for file

Additional file 2 **Effects of si-RNA treatments on oxidative stress and AAH immunoreactivity**. Figure depicting immunohistochemical staining for 4-hydroxy-2-nonenal and aspartyl-(asparaginyl)-β-hydroxylase (AAH) in cerebella of P16 rats that, on P2, were administered ICV injections of (A,E) si-Scr, (B,F) si-InR, (C,G) si-IGF-1R, or (D,H) si-IGF-2R. Paraffin-embedded histological sections of cerebella from P16 rats that were administered ICV injections of (A,E) si-Scr, (B,F) si-InR, (C,G) si-IGF-1R, or (D,H) si-IGF-2R on P2 were immunostained to detect (A-D) HNE as an index of oxidative stress and lipid peroxidation, or (E-H) AAH, a gene regulated by insulin/IGF stimulation and a mediator of neuronal migration during development. Immunoreactivity was detected with HRP-conjugated polymer-linked secondary antibody and DAB as the chromogen (brown). Insets show higher magnification images of the granule cell/Purkinje cell layers. Note minimal HNE immunoreactivity in the si-Scr and si-IGF-1R treated brains, and increased labeling of both granule (small round nuclei) and Purkinje (pyramid or star-shaped) cells in the si-InR and si-IGF-2R treated brains. In contrast, AAH immunoreactivity was robust in all cortical layers of si-Scr, si-InR, and si-IGF-1R treated brains, and reduced in the granule cell layers of si-IGF-2R treated brains. In addition, in the si-InR treated brains, Purkinje cells exhibited intense AAH immunoreactivity (F). Original magnifications, 100×; insets, 400×. Click here for file
