# Supplementary material for: si-RNA inhibition of brain insulin or insulin-like growth factor receptors causes developmental cerebellar abnormalities: relevance to fetal alcohol spectrum disorder
Source: Mol Brain. 2011 Mar 28;4:13. doi: 10.1186/1756-6606-4-13 (PMC3077327; doi:10.1186/1756-6606-4-13)
Supplement: Additional file 1 — Primer pairs used for qRT-PCR assays. The table includes primer pair sequences, primer binding positions on the mRNA, and the amplicon (PCR product) sizes. [file 1756-6606-4-13-S1.DOC]

Additional File 1: Primer pairs used for qRT-PCR assays

| Primer | Direction | Sequence (5’🡪3’) | Position (mRNA) | Amplicon Size (bp) |
| --- | --- | --- | --- | --- |
| 18S rRNA | For | GGA CAC GGA CAG GAT TGA CA | 1278 | 50 |
| 18S rRNA | Rev | ACC CAC GGA ATC GAG AAA GA | 1327 |  |
| Insulin | For | TTC TAC ACA CCC AAG TCC CGT C | 145 | 135 |
| Insulin | Rev | ATC CAC AAT GCC ACG CTT CTG C | 279 |  |
| Insulin Receptor | For | TGA CAA TGA GGA ATG TGG GGA C | 875 | 129 |
| Insulin Receptor | Rev | GGG CAA ACT TTC TGA CAA TGA CTG | 1003 |  |
| IGF-1 | For | GAC CAA GGG GCT TTT ACT TCA AC | 65 | 127 |
| IGF-1 | Rev | TTT GTA GGC TTC AGC GGA GCA C | 191 |  |
| IGF-1 Receptor | For | GAA GTC TGC GGT GGT GAT AAA GG | 2138 | 113 |
| IGF-1 Receptor | Rev | TCT GGG CAC AAA GAT GGA GTT G | 2250 |  |
| IGF-2 | For | CCA AGA AGA AAG GAA GGG GAC C | 763 | 95 |
| IGF-2 | Rev | GGC GGC TAT TGT TGT TCA CAG C | 857 |  |
| IGF-2 Receptor | For | TTG CTA TTG ACC TTA GTC CCT TGG | 1066 | 91 |
| IGF-2 Receptor | Rev | AGA GTG AGA CCT TTG TGT CCC CAC | 1156 |  |
| BDNF | For | AAC TCG CAA TGC CGA ACT ACC | 1279 | 92 |
| BDNF | Rev | GTG TCT ATC CTT ATG AAC CGC CAG | 1370 |  |
| NGFβ | For | TTG GAG ATA AGA CCA CAG CCA CGG | 683 | 69 |
| NGFβ | Rev | TAA TGT TCA CCT CGC CCA GCA CTG | 751 |  |
| P75-NTR | For | TGC TTG CTG TTG GAA TGA GTG G | 862 | 78 |
| P75-NTR | Rev | ACA ATG CTC CTG GTC TCT TCA CC | 939 |  |
| NRTK | For | GGA GTT GAG AAG CCT AAC CAT CG | 334 | 104 |
| NRTK | Rev | CAT TGG AGG AGA GAT TCA GGT GAC | 437 |  |

*Abbreviations: IGF=insulin-like growth factor; BDNF=Brain derived neurotrophic factor; P75-NTR= P75 neurotrophin receptor; NRTK= neuronal tyrosine kinase receptor; NGF= nerve growth factor; bp= base pair.
