# Supplementary figures and images for: si-RNA inhibition of brain insulin or insulin-like growth factor receptors causes developmental cerebellar abnormalities: relevance to fetal alcohol spectrum disorder
Source: Mol Brain. 2011 Mar 28;4:13. doi: 10.1186/1756-6606-4-13 (PMC3077327; doi:10.1186/1756-6606-4-13)

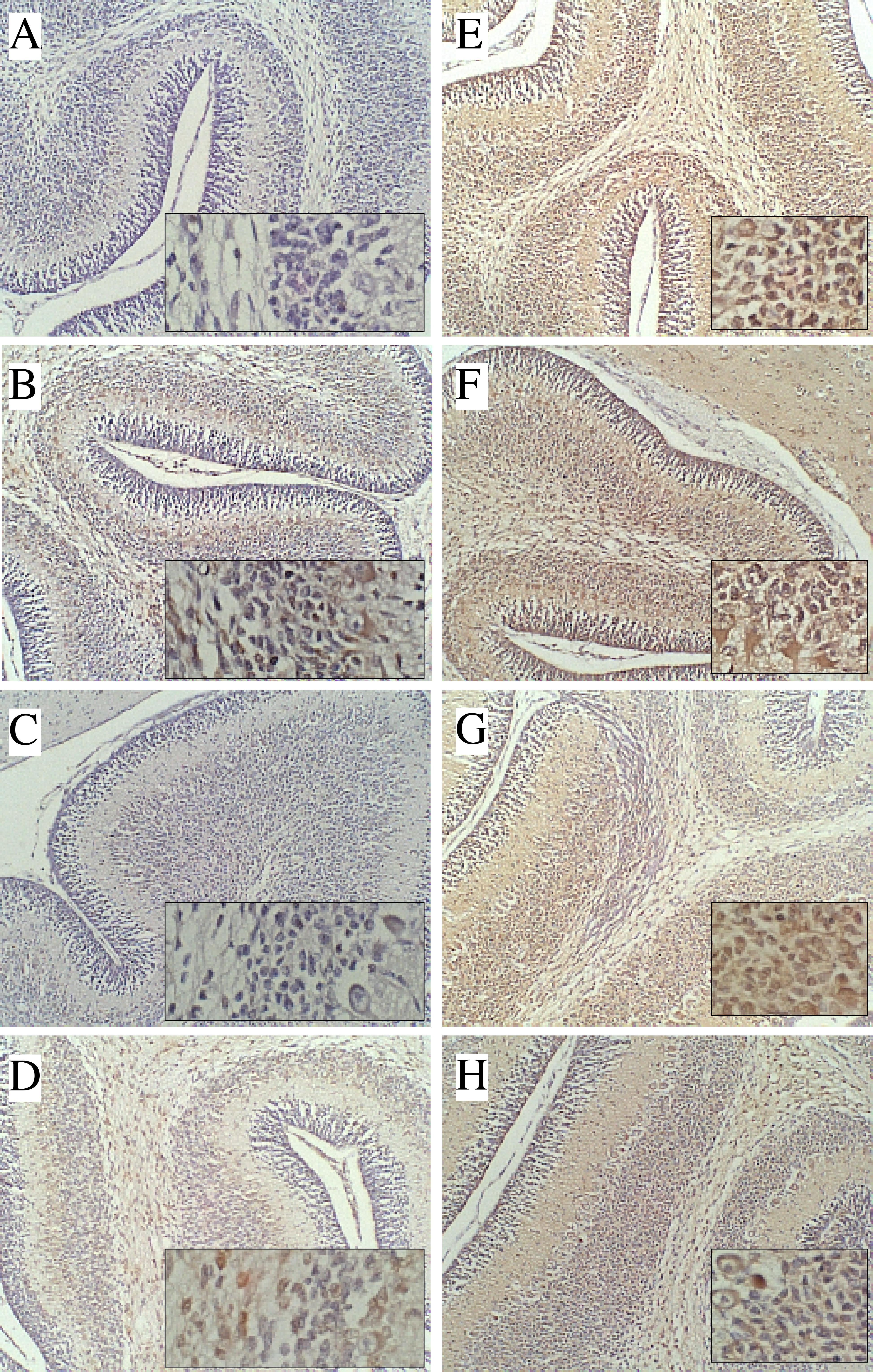

Supplement: Additional file 2 — Effects of si-RNA treatments on oxidative stress and AAH immunoreactivity. Figure depicting immunohistochemical staining for 4-hydroxy-2-nonenal and aspartyl-(asparaginyl)-β-hydroxylase (AAH) in cerebella of P16 rats that, on P2, were administered ICV injections of (A,E) si-Scr, (B,F) si-InR, (C,G) si-IGF-1R, or (D,H) si-IGF-2R. Paraffin-embedded histological sections of cerebella from P16 rats that were administered ICV injections of (A,E) si-Scr, (B,F) si-InR, (C,G) si-IGF-1R, or (D,H) si-IGF-2R on P2 were immunostained to detect (A-D) HNE as an index of oxidative stress and lipid peroxidation, or (E-H) AAH, a gene regulated by insulin/IGF stimulation and a mediator of neuronal migration during development. Immunoreactivity was detected with HRP-conjugated polymer-linked secondary antibody and DAB as the chromogen (brown). Insets show higher magnification images of the granule cell/Purkinje cell layers. Note minimal HNE immunoreactivity in the si-Scr and si-IGF-1R treated brains, and increased labeling of both granule (small round nuclei) and Purkinje (pyramid or star-shaped) cells in the si-InR and si-IGF-2R treated brains. In contrast, AAH immunoreactivity was robust in all cortical layers of si-Scr, si-InR, and si-IGF-1R treated brains, and reduced in the granule cell layers of si-IGF-2R treated brains. In addition, in the si-InR treated brains, Purkinje cells exhibited intense AAH immunoreactivity (F). Original magnifications, 100×; insets, 400×. [file 1756-6606-4-13-S2.JPEG]
